# Trends in inpatient discharges with drug or alcohol admission diagnoses to a skilled nursing facility among older adults, New York City 2008–2014

**DOI:** 10.1186/s12954-020-00450-8

**Published:** 2020-12-10

**Authors:** Benjamin H. Han, Ellenie Tuazon, Hillary V. Kunins, Denise Paone

**Affiliations:** 1 Division of Geriatrics and Gerontology, Department of Medicine, University of California San Diego School of Medicine, La Jolla, CA 92093 USA; 2 Bureau of Alcohol and Drug Use Prevention, Care, and Treatment, New York City Department of Health and Mental Hygiene, 42-09 28th Street, 19th Floor, Queens, NY 11101 USA

**Keywords:** Post-acute care, Long-term care, Substance use, Geriatrics

## Abstract

**Background:**

Recent research shows an increase in drug and alcohol-related hospitalizations in the USA, especially among older adults. However, no study examines trends in discharges to a skilled nursing facility (SNF) after a drug or alcohol-related hospitalization. Older adults are more likely to need post-hospital care in a SNF after a hospitalization due to an increased presence of chronic diseases and functional limitations. Therefore, the objective of this study was to estimate trends in drug or alcohol-related hospitalizations with discharge to a SNF among adults age 55 and older.

**Methods:**

We analyzed data from New York State’s Statewide Planning and Research Cooperative System to calculate the number of cannabis, cocaine, opioid, and alcohol-related hospitalizations in New York City that resulted in discharge to a SNF from 2008 to 2014 among adults age 55 and older. Using New York City population estimates modified from US Census Bureau, we calculated age-specific rates per 100,000 adults. Trend tests were estimated using joinpoint regressions to calculate annual percentage change (APC) with 95% confidence intervals (CI) and stratified by adults age 55–64 and adults age 65 and older.

**Results:**

During the study period, among adults age 55–64, there were significant increases in cocaine, cannabis, and opioid-related hospitalizations that resulted in discharge to a SNF. For adults ≥ 65 years, there were sharp increases across all substances with larger increases in opioids (APC of 10.66%) compared to adults 55–64 (APC of 6.49%). For both age groups and among the four substances, alcohol-related hospitalizations were the leading cause of discharge to a SNF.

**Conclusions:**

We found an increase in hospital discharges to SNFs for patients age 55 and older admitted with alcohol or drug-related diagnoses. Post-acute and long-term care settings should prepare to care for an increase in older patients with substance use disorders by integrating a range of harm reduction interventions into their care settings.

## Background

Older adults with substance use disorder (SUD) are often medically complex with the presence of multiple chronic conditions [1, 2] and are especially vulnerable to the harms of drug and alcohol use including overdose [3]. Furthermore, substance use itself can exacerbate existing chronic medical disease [1]. It is therefore not surprising that we are seeing large increases in alcohol-related [4] and opioid-related hospitalizations among older adults in the USA [5, 6]. The intersection of aging, chronic medical diseases, and substance use is complex [7]. Older adults have more chronic health conditions, functional impairments, and are more likely to be hospitalized [8], which increases the likelihood that older adults with SUDs will need post-acute care after hospitalization. Older adults may be hospitalized primarily for a drug and alcohol-related problem but also have an exacerbation of a chronic medical disease. Alternatively, older adults may have a SUD that is stable (e.g., taking methadone or buprenorphine for opioid use disorder for many years) but hospitalized for an acute medical problem. Increases in hospitalizations among older adults with SUDs have implications for the US healthcare system. These include a higher demand for SUD care in settings that have traditionally not seen high rates of patients with SUDs, including post-acute and long-term care settings.

Skilled nursing facilities (SNFs) provide skilled nursing care for short-term post-hospital care (e.g., intravenous therapy, wound care, physical, and occupational therapy) as well as providing long-term care for certain patients (e.g., patients living with dementia). SNFs serve an important role in the US healthcare system by caring for patients who no longer require inpatient care, but require further rehabilitation services, nursing care, or chronic disease management to improve function and return home. Medicare Part A covers SNF care after a qualifying hospital stay for up to 100 days. Despite the sharp increases in drug or alcohol-related hospitalizations among older adults [4–6] there remain little data on the prevalence of such patients entering SNFs after hospitalization for post-acute care. While high rates of patients with SUDs have been noted and characterized in the US Department of Veterans Affairs (VA) nursing homes, mainly for alcohol use disorders [9, 10], there are few data on SUDs in non-VA long-term or post-acute care settings [11]. In addition, there is a lack of studies that examine SUD treatment in post-care settings since the start of the current opioid epidemic. Unfortunately, individuals with SUDs such as opioid use disorder may be excluded from many post-acute care settings such as SNFs for a range of reasons including stigma and logistical or regulatory issues of providing methadone or buprenorphine for opioid use disorder [12, 13]. For this study we analyzed inpatient data in New York City to examine trends in hospital discharges to SNF admissions for patients who were hospitalized with an admission diagnosis of SUD, acute drug poisoning, or health condition that is a direct result of alcohol use. This research is imperative to understand post-acute care utilization among adults with SUD, which is likely to involve older adults with more medical comorbidity.

## Methods

We used data available from the Statewide Planning and Research Cooperative System (SPARCS), a New York payer data reporting system that collects patient-level data from inpatient hospital discharge. SPARCS contains patient-level information on all hospital discharges and ED admissions in New York State, including primary and secondary admission diagnoses and discharge disposition [14]. For this study we utilized inpatient discharges from the full calendar years from 2008 to 2014 for all New York City hospitals.

### Measures

We identified all inpatient hospitalizations with an admission diagnosis code (either primary or secondary diagnoses) with ICD-9 codes related to opioids (opioid abuse, opioid dependence, opioid poisoning, or heroin poisoning), cocaine (abuse, dependence, or poisoning), cannabis (abuse or dependence), and alcohol (dependence syndrome, abuse, withdrawal, toxic effect of ethyl alcohol, and alcohol-related pellagra, polyneuropathy, cardiomyopathy, gastritis, hepatitis, and liver damage). We excluded hospitalizations that were specific for detoxification or drug/alcohol rehabilitation to focus on inpatient medical hospitalizations and excluded ICD-9 codes that involved suicide and self-inflicted poisoning or assault. We classified each hospitalization based on a SPARCS-coded patient status or disposition (discharge status) and identified the number of opioid, cocaine, cannabis, and alcohol-related hospitalizations with a disposition status of “discharged/transferred to Skilled Nursing Facility (SNF) with Medicare Certification in Anticipation of Skilled Care”). This study was exempted from institutional board review as it was considered public health surveillance.

### Statistical analysis

We used Joinpoint Regression software (Centers for Disease Control, version 4.5.01) to examine trends between 2008 and 2014 in alcohol and drug-related hospitalizations to SNFs. Joinpoint regression modeling techniques detect increases or decreases between points which are known as joinpoints. These joinpoints are evaluated using permutations that look for significant changes in linear trends between points [15]. To identify trends, we applied the grid-search method, which sets up the data points in a grid to search for all possible joinpoints. We specified a minimum of zero joinpoints (one singular trend) to a maximum of two joinpoints due to the number of years under review (7 years of data from 2008 through 2014). We used the Monte Carlo permutation with an overall significance level of 0.05. For any identified trends, the change in slope of a linear trend was described as the annual percent change (APC). We used joinpoint regression to see whether multiple trends were present for each substance examined.

We used the 2000 US standard and New York City population estimates, modified from US Census Bureau intercensal population estimates by year from 2008 to 2014, to calculate yearly, age-specific rates per 100,000 people. We reported the trends by substance type (alcohol, cannabis, cocaine, and opioids) and age group (55–64 and ≥ 65 years) using annual percent change (APC), 95% confidence intervals, and *p* values, which identified whether the annual percent change differed from zero. We described these as percent increase or decrease per year and reported statistically significant (*p* < 0.05) findings.

## Results

**Fig. 1:**
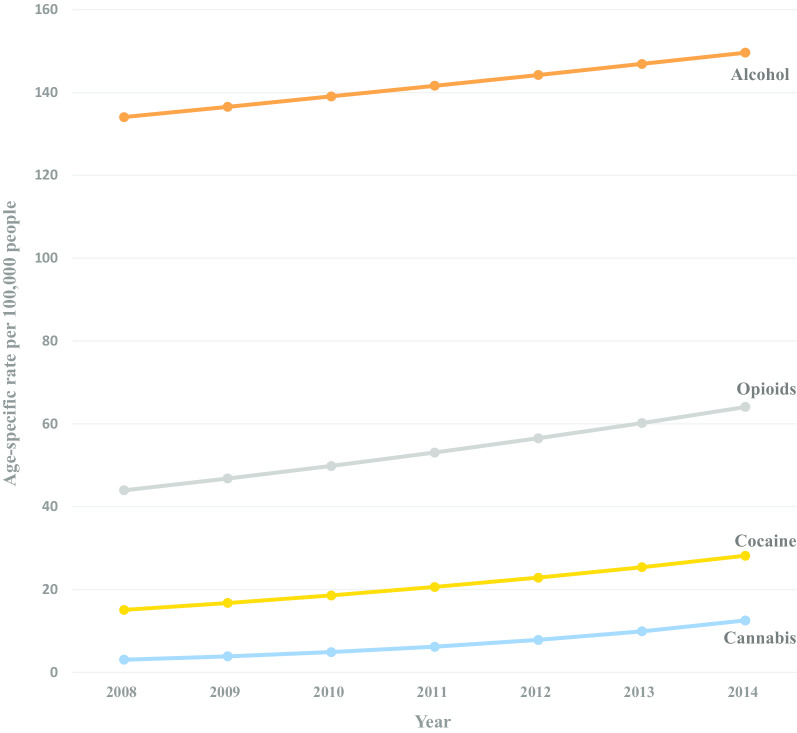
Trends in hospital discharges with a drug or alcohol admission diagnosis to a skilled nursing facility (SNF), adults age 55–64, New York City 2008–2014

**Fig. 2:**
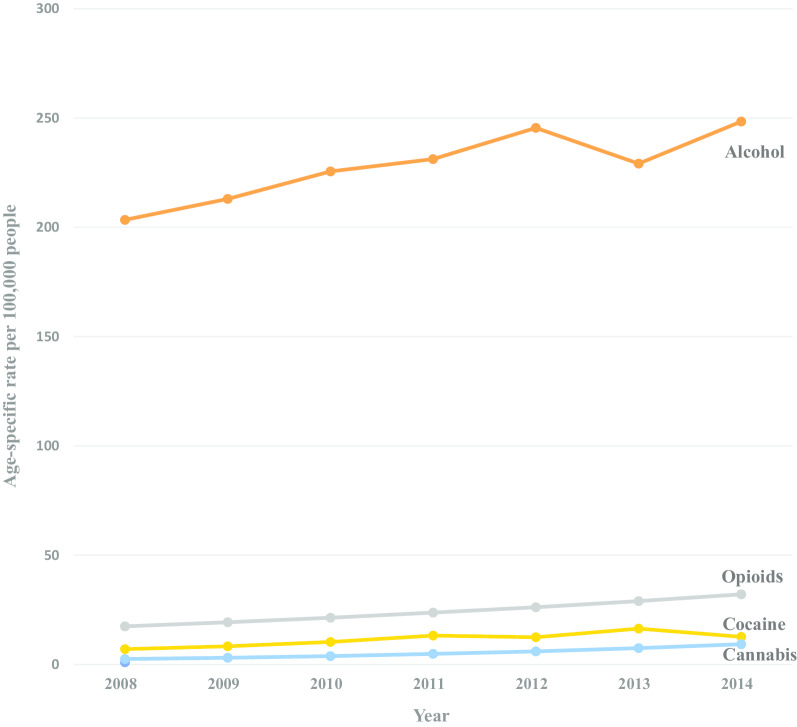
Trends in hospital discharges with a drug or alcohol admission diagnosis to a skilled nursing facility (SNF), adults age 65 and older, New York City 2008–2014

From 2008 through 2014, we identified 699,479 total opioid, cocaine, cannabis, or alcohol-related hospitalizations among patients age 55 and older with 40,307 of those hospitalizations resulting in a discharge to a SNF. During the study period, discharges to SNFs increased among hospitalizations with diagnoses of alcohol, opioid, cocaine, and cannabis for both adults age 55–64 and ≥ 65 years (Figs. 1, 2), all with singular trends. Across all years, adults 55–64 had higher rates per 100,000 for all substances compared to adults ≥ 65 years (Table 1). Cannabis-related hospitalizations with discharges to SNFs had the largest annual percentage increase for both age groups, with an APC of 26.55% for those 55–64, and an APC of 24.42% for adults ≥ 65 years. Opioid-related admissions with discharges to SNFs also increased for both age groups, but there was a larger increase among those ≥ 65 (APC of 10.66%, *p* < 0.001) compared to those 55–64 years (APC of 6.49%, *p* = 0.003). Both age groups had similar significant annual increases for cocaine-related admissions with discharges to SNFs among adults age 55–64 having an APC of 10.98%, *p* = 0.01, and those ≥ 65 having an APC of 11.81%, *p* = 0.02. For alcohol-related hospital admissions with discharges to SNFs, the 55–64 group did not experience a statistically significant annual increase (an APC of 1.85%, *p* = 0.17). While those 65 and older saw a dip in alcohol-related hospital admissions with discharges to SNFs from 2012 to 2013, there was an overall significant increase between 2008 and 2014 with an APC of 2.96%, *p* = 0.01.

**Table 1:** Annual percentage changes in hospital discharges with a drug or alcohol diagnosis to a skilled nursing facility (SNF), New York City, by age group

	Time period	Annual percentage change	95% confidence interval	*p* value
*Age 55–64*				
Alcohol	2008–2014	1.85	− 1.12, 4.90	0.17
Cocaine	2008–2014	10.98	3.58, 18.90	0.01
Cannabis	2008–2014	26.55	15.90, 38.18	0.001
Opioids	2008–2014	6.49	3.33, 9.74	0.003
*Age 65 and older*				
Alcohol	2008–2014	2.96	1.18, 4.77	0.01
Cocaine	2008–2014	11.81	2.62, 21.82	0.02
Cannabis	2008–2014	24.42	7.29, 44.29	0.01
Opioids	2008–2014	10.66	8.07, 13.32	< 0.001

## Discussion

This is the first study to our knowledge that examines trends in hospital discharges to skilled nursing facilities for patients initially admitted for drug or alcohol reasons. We found increases for alcohol, cocaine, cannabis, and opioid-related hospitalizations that resulted in discharge to a SNF in New York City among adults age 55 and older. SNFs play a critical role in reducing healthcare-related costs by decreasing length of hospitalizations, the rehabilitation of patients through physical and occupational therapy who are not yet functional to return home, and by optimizing chronic disease management for patients. However, there is sparse literature on how SNFs in the USA manage patients with SUD. As substance use increases among middle-aged and older adults with chronic medical diseases [1, 7], along with increases in drug and alcohol-related hospitalizations [4, 5], SNFs will also need to be prepared to care for adults with chronic medical disease who have a history of SUD.

Several barriers exist for providing treatment of SUDs in SNFs. Methadone and buprenorphine are evidence-based treatments for opioid use disorder that reduces all cause and overdose mortality [16]. There are many logistical issues with supplying methadone for opioid use disorder in healthcare facilities that are not registered as a federally licensed opioid treatment program including nearly all SNF [12, 17]. Furthermore, only approximately 5% of medical providers are licensed to prescribe buprenorphine [18]. Meanwhile, the findings in our study show sharp increases in opioid-related hospitalizations resulting in discharge to a SNF, particularly among patients 65 and older. This indicates that SNFs will need to build capacity and work out logistical concerns to be able to provide methadone or buprenorphine for opioid use disorder in the post-acute and long-term care settings. Additionally, SNFs could also be an ideal place to review opioid overdose prevention with naloxone training for patients who may have been admitted for prescription opioid poisonings or overdose and their families. With the federal restrictions limiting the ability to prescribe methadone for opioid use disorder, efforts should be made to increase the number of providers working in SNFs who have their waiver to prescribe buprenorphine. These constraints should not preclude patient access to evidence-based treatment for a chronic medical disease. Therefore, as more adults age 55 and older take medications for opioid use disorder [19], SNFs will need the capacity to continue SUD care whether a patient’s disease is stable or not. A broad effort should be made to increase the number of medical providers working in SNFs with a buprenorphine Drug Addiction Treatment Act (DATA) X waiver either from targeted training or discontinuing the buprenorphine X waiver to reduce the barriers to prescribing [20]. We also recognize the multiple challenges that exist in providing quality care in SNF settings for complex medical patients before even considering SUDs [21] and, therefore, models of care need to be developed to help assist providers in SNFs with the management of patients with SUD.

The study results also show particularly sharp increases in cannabis and cocaine-related hospitalizations resulting in discharge to SNFs among adults age 55 older. The increase in cannabis use in the USA, including among older adults [22], has resulted in an increase in cannabis use disorders [23] and increases in cannabis-related emergency department visits and hospitalizations [24, 25]. While there is increasing interest in medical cannabis for the treatment of chronic disease and symptoms for older adults in SNFs [26], our study focuses on cannabis use disorder and, therefore, problematic cannabis use, for which treatment is primarily psychotherapy [27]. SNFs in many states therefore will need to be aware of both the increased demand for medical cannabis in their facility while also experiencing an increase in patients who have cannabis use disorder who may need access to psychotherapy treatments. In addition, New York City has experienced high rates of cocaine-related emergency department visits and hospitalizations, especially among adults age 45–64 [28], and cocaine-related overdose deaths have increased sharply among adults age 55 and older from 2009 to 2016 [3]. While there are no FDA-approved medication treatments for cannabis or cocaine use disorder (unlike opioid use disorder), SNFs should be prepared to offer resources such as cognitive-behavioral therapy, motivational interviewing, peer recovery coaches, harm reduction education, and resources in the community for patients who may benefit. Finally, while alcohol-related hospital admissions to SNFs had a less pronounced increase from 2008 to 2014 in our analysis, there was a significant annual increase among adults age 65 and older. Alcohol-related hospitalizations were also overwhelmingly more common than cocaine, cannabis, and opioid-related hospitalizations in our study. More data exist for patients with alcohol use disorders in SNFs [9, 10], including treatment in this setting [29]. Treatment with naltrexone for patients admitted for alcohol use disorder can be started during hospitalization [30] and continued in the post-acute care setting without logistical issues.

With the increase in middle-aged and older adults with SUDs [7], the post-acute and long-term care settings present an opportunity to expand and optimize treatment for SUDs along with other chronic medical diseases after an acute hospitalization. The priority should be to ensure that all Medicare-certified SNFs are able to provide evidence-based medications, therapy, and counseling for patients with substance use disorders. A possible solution to reduce the burden for SNF staff who do not have expertise in substance use disorder treatment would be to develop addiction medicine consultant services, similar to ones developed for inpatient hospital settings [31]. These multidisciplinary teams could see patients with SUDs in the post-acute setting in SNFs. Such a consultant or team could work in tandem with SNF providers to assist in transitions of care from the inpatient setting to SNFs to ensure continuation of methadone or buprenorphine if indicated and provide optimization of care for SUD in SNFs with addiction medicine expertise, behavioral interventions, and peer counseling.

### Limitations

This paper has several important limitations to consider in the interpretation of our results. First, our SPARCS datasets have only admitting diagnoses and not discharge diagnoses; therefore, it is possible that the admission diagnoses may be initially incorrect or differ from the discharge diagnoses. This could potentially underestimate or overestimate our prevalence estimates. Furthermore, an individual could have a SUD that is stable and be admitted for a different medical reason, and the admitting provider did not list the SUD as one of the admitting diagnoses and thus not captured in our study. Also, this study does not distinguish between a patient with an acute substance-related issue (i.e., overdose) versus a patient with a stable SUD. While these patients would likely have different clinical management strategies, both will likely still need to have their substance-related problems managed, whether as an acute disease or a chronic one. Next, we examined all hospitalizations resulting in discharge to SNFs and not by individuals; therefore, a small group of individuals could drive up the prevalence rate with multiple readmissions to the hospital; however, in sensitivity analysis < 3% of our samples was due to individuals with multiple admissions. In addition, due to the implementation of the ICD-10-CM classification in October 2015, we are unable to provide more recent trends given that the different classification system prevents direct comparisons [32]. Future analysis of alcohol and drug-related discharges to SNFs after October 2015 can provide more recent trend information.

We also limited our analysis to adults age 55 and older given the relatively low prevalence of discharge to SNF for adults younger than 55 years of age. Finally, our analysis is limited to New York City hospitals and therefore not generalizable to the rest of the USA.

## Conclusions

Older adults have more chronic health conditions and functional impairments and are more likely to be hospitalized, which increases the likelihood that those with SUDs will need post-acute care after hospitalization. In New York City from 2008 to 2014, there were increases in alcohol and drug-related hospitalizations that resulted in a discharge to a SNF among adults age 55 and older. Skilled nursing facilities must prepare to care for a growing population of older adults with substance use problems. A range of evidence-based harm reduction interventions to treat patients with substance use disorders should be introduced into the post-acute and long-term care settings.

## Data Availability

The data used are from the Statewide Planning and Research Cooperative System (SPARCS), which are available at: https://www.health.ny.gov/statistics/sparcs/access/.

## Abbreviations

APC: Annual percent change
SNF: Skilled nursing facility
SPARCS: Statewide Planning and Research Cooperative System
SUD: Substance use disorder
VA: Veterans affairs
US: USA
